# Bayesian Gaussian distributional regression models for more efficient norm estimation

**DOI:** 10.1111/bmsp.12206

**Published:** 2020-07-20

**Authors:** Lieke Voncken, Thomas Kneib, Casper J. Albers, Nikolaus Umlauf, Marieke E. Timmerman

**Affiliations:** ^1^ Department of Psychometrics & Statistics Faculty of Behavioural and Social Sciences University of Groningen The Netherlands; ^2^ Department of Methodology and Statistics Tilburg School of Social and Behavioral Sciences Tilburg University The Netherlands; ^3^ Department of Statistics and Econometrics Faculty of Business and Economics Georg‐August‐Universität Göttingen Germany; ^4^ Department of Statistics Faculty of Economics and Statistics Universität Innsbruck Austria

**Keywords:** BAMLSS, continuous test norming, norming efficiency, psychological tests, robustness

## Abstract

A test score on a psychological test is usually expressed as a normed score, representing its position relative to test scores in a reference population. These typically depend on predictor(s) such as age. The test score distribution conditional on predictors is estimated using regression, which may need large normative samples to estimate the relationships between the predictor(s) and the distribution characteristics properly. In this study, we examine to what extent this burden can be alleviated by using prior information in the estimation of new norms with Bayesian Gaussian distributional regression. In a simulation study, we investigate to what extent this norm estimation is more efficient and how robust it is to prior model deviations. We varied the prior type, prior misspecification and sample size. In our simulated conditions, using a fixed effects prior resulted in more efficient norm estimation than a weakly informative prior as long as the prior misspecification was not age dependent. With the proposed method and reasonable prior information, the same norm precision can be achieved with a smaller normative sample, at least in empirical problems similar to our simulated conditions. This may help test developers to achieve cost‐efficient high‐quality norms. The method is illustrated using empirical normative data from the IDS‐2 intelligence test.

## 1 Introduction

Psychological tests are widely used to assess individuals in clinical and educational contexts. Such tests are designed to measure, for instance, an individual's developmental level, intelligence or ability level. The scores on these tests are usually interpreted relative to the scores of the reference population, while the reference population may depend on individual characteristic(s). For example, the reference population for intelligence tests is typically the general population in the same country and of the same age as the testee involved, and for neuropsychological tests the healthy population in the same country, of the same age, gender and educational level as the testee. A normed score is a transformed version of a raw score. Normed scores can be expressed in various ways, such as percentiles, (normalized) *z*‐scores or IQ scores (Mellenbergh, 2011, pp. 351–357). Transformation rules are estimated during the test construction phase, based on test scores from a normative sample. This sample represents the reference population, possibly conditional upon relevant individual characteristic(s).

When norms depend on individual characteristic(s), such as age, this implies that one has multiple reference populations. For age‐dependent norms, the number of reference populations is (strictly speaking) infinite, as there is one for each specific age within the age range of the test. Traditionally, such norms were derived from the empirical raw test score distributions within subgroups of (combinations of) the relevant individual characteristic(s), such as age groups (e.g., in the Wechsler Intelligence Scale for Children–III (WISC‐III); Wechsler, 1991). It was (implicitly) assumed that the test score distributions are equal for all ages within a subgroup, and that this distribution changes as a step function of continuous variable(s) involved. This assumption is typically unrealistic, and then it makes sense to assume that the relationship between continuous variable(s) and the test distribution is smooth (Van Breukelen & Vlaeyen, 2005; Zachary & Gorsuch, 1985). Such a smooth function could be approximated better by making the subgroups smaller. Yet, this would increase the sampling variability in the estimated norms, as fewer observations per subgroup would be available to estimate the raw test score distribution. These issues are circumvented in continuous norming (Zachary & Gorsuch, 1985), in which the test score distribution is estimated as a continuous function of the predictor(s) in a regression model. Continuous norming is more efficient than traditional norming (Oosterhuis, van der Ark, & Sijtsma, 2016), because all observations in the normative sample are used jointly to estimate the raw test score distribution, rather than only the observations within a subgroup.

There are three main continuous norming approaches: inferential norming (Wechsler, 2008; Zachary & Gorsuch, 1985; Zhu & Chen, 2011), nonparametric norming (Lenhard, Lenhard, & Gary, 2019; Lenhard, Lenhard, Suggate, & Segerer, 2018; Tellegen & Laros, 2014) and moments regression‐based norming (Oosterhuis, 2017; Van Breukelen & Vlaeyen, 2005; Voncken, Timmerman, Spikman, & Huitema, 2018). In inferential norming, moments of the raw test score distributions are computed for subgroups of the normative sample, and these moments are regressed on subgroup‐level predictor(s). The advantage of this continuous norming approach is that it does not require strong assumptions on the shape of the conditional test score distribution. The disadvantage is that the moments are estimated for each subgroup, which could reduce the precision and efficiency of the estimates, and could result in biased estimates – as they depend on the exact subgroups used.

In nonparametric norming, the relationship of the raw test scores with the normed scores and age is modelled using regression involving Taylor polynomials. The advantage of this approach is that it does not require any assumptions about the shape of the conditional score distribution. The disadvantages are that the resulting percentile curves can intersect, which is impossible from a theoretical point of view, and that it requires discretizing the continuous predictor variable to estimate the normed scores, just as in inferential norming. Thus, the results may be biased.

In moments regression‐based norming, moments of interest are regressed on predictor(s) for individual raw test score data, rather than for subgroup data. Van Breukelen and Vlaeyen (2005) and Oosterhuis (2017) used a standard regression model to estimate the mean of the raw test score distribution conditional on the predictor(s). This approach does not require discretization of the predictor variable(s) at all, and is guaranteed to yield non‐intersecting percentile curves. However, using a standard regression model assumes normality of the conditional raw test score distributions, with a constant variance. This is often an unrealistic assumption, as the assumptions of normality and homoscedasticity are rarely fulfilled in psychometric tests (e.g., Lenhard *et al*., 2019). For instance, a floor effect expresses itself in skewness of the test score distribution. That is why we use a more flexible moments regression‐based norming approach – via distributional regression – that allows for modelling heteroscedasticity and non‐normality. In this approach, the distributional characteristics are estimated as functions of the predictor(s). For example, the mean, standard deviation and skewness of the test score can vary conditional on age. A frequentist distributional regression framework (i.e., generalized additive models for location, scale and shape (GAMLSS); Rigby & Stasinopoulos, 2005) has successfully been applied to estimate normed scores for different types of psychological tests (e.g., developmental tests, intelligence tests and neuropsychological tests; Bayley, 2006; Grob & Hagmann‐von Arx, 2018; Rommelse *et al*., 2018; Voncken, Albers, & Timmerman, 2019; Voncken *et al*., 2018). The normed scores of these tests are estimated conditional on age, and sometimes (i.e., in neuropsychological tests) also conditional on the additional predictors sex and/or education level.

The flexibility of distributional regression allows for precise distribution estimation. Yet, this flexibility can result in complex models that require a large sample to estimate the parameters with sufficient expected precision. As it is very time‐consuming and expensive – and not always possible in practice – to collect a large normative sample, we aim to make norm estimation more efficient by incorporating prior information in the estimation of new norms. To do this, we apply Bayesian distributional regression in the context of continuous norming. Although this approach can be applied to many different models, we focus on Gaussian distributional regression models in this paper as a proof of concept.

Using a Bayesian approach in norming has two main advantages. First, it allows us to take into account prior information in the norming process. In the norming context, a reasonable informative prior can be derived from normative sample data of the same test in a different country, or from older norms. The latter are often available as norms can become outdated (Wasserman & Bracken, 2013) and renorming is warranted. Second, it allows us to estimate and collect normative data in an iterative way. This implies that one can stop sampling when the desired level of norm precision is achieved.

The remainder of this paper is structured as follows. First, we will briefly discuss Bayesian distributional regression and how this can be used to include prior norm information in a new norming model. Second, we will assess in a simulation study how much efficiency is gained and how robust Bayesian distributional regression is with respect to prior misspecification. Third, we will illustrate the procedure of including prior norm information with empirical normative data from an intelligence test. Finally, we will discuss the results and implications.

## 2 Bayesian Gaussian distributional regression

In Gaussian distributional regression models, the explanatory variables are related to the mean and standard deviation of the distribution as follows:

$$y_{i}|x_{i}∼D\left(h_{\mu }\left(\theta_{\mu }\left(x_{i}\right)\right)=\eta_{i\mu },h_{\sigma }\left(\theta_{\sigma }\left(x_{i}\right)\right)=\eta_{i\sigma }\right),$$

where D denotes the parametric distribution for the response variable $y_{i}$ for observation *i* (*i* = 1, …, *N*), with distributional parameters θ*_k_* (*k* = µ, σ) for the mean and standard deviation, respectively, that are related to the covariate observations for observation *i*, $x_{i}$. This can be generalized to other (i.e., non‐Gaussian) distributions by using additional, and possibly different, distributional parameters θ*_k_*. The distributional parameters $\theta_{k}\left(x_{i}\right)$ are linked to the additive predictors $\eta_{\mathrm{ik}}$ using link functions $h_{k}\left(\cdot \right)$, which ensure that only admissible values for the distributional parameters can be observed (e.g., non‐negative variances).

The *k*th additive predictor is given by

$$\eta_{\mathrm{ik}}=f_{1k}\left(x_{i};\beta_{1k}\right)+\ldots +f_{J_{k}k}\left(x_{i};\beta_{J_{k}k}\right),$$

where the functions $f_{\mathrm{jk}}\left(\cdot \right)$, $j=1,\ldots ,J_{k}$, relate to the regression effect as characterized by regression parameters $\beta_{\mathrm{jk}}$. Smooth nonlinear relationships between the distributional parameters and predictor(s) can be modelled using polynomials or splines. The disadvantage of polynomials is that values of observed scores conditional on a certain predictor value might have a large and undesirable influence on the predicted score at a very different value of the predictor (Magee, 1998). Splines do not have this problem, because they operate more locally than polynomials. In this paper, we therefore use splines. Specifically, we use so‐called P‐splines, which are penalized B‐splines (Eilers & Marx, 1996, 2010). The advantage of P‐splines, unlike for example (non‐penalized) B‐splines, is that they are numerically stable, easy to implement, and allow for varying the degree of smoothing with only a single parameter (Eilers & Marx, 1996).

In Bayesian Gaussian distributional regression, prior information is embedded in the prior $p_{\mathrm{jk}}\left(\cdot \right)$ of the *jk*th model term. The posterior is proportional to the likelihood times the prior. For computational simplicity, the log‐posterior

$$\begin{array}{c} \log\pi \left(\beta ,\tau ;y,X,\alpha \right)\propto \ell \left(\beta ;y,X\right)+\sum_{k=1}^{K}\sum_{j=1}^{J_{k}}\left[\log p_{\mathrm{jk}}\left(\beta_{\mathrm{jk}};\tau_{\mathrm{jk}},\alpha_{\mathrm{jk}}\right)\right] \end{array}$$

is used, where $\tau_{\mathrm{jk}}$ are the smoothing variances that regulate the importance of the prior relative to the likelihood, $\alpha_{\mathrm{jk}}$ are the fixed prior specifications, and $\ell \left(\beta ;y,X\right)$ is the log‐likelihood function. The prior for the *jk*th model term is given by

$$\begin{array}{c} p_{\mathrm{jk}}\left(\beta_{\mathrm{jk}};\tau_{\mathrm{jk}},\alpha_{\mathrm{jk}}\right)\propto d_{\beta_{\mathrm{jk}}}\left(\beta_{\mathrm{jk}}|\tau_{\mathrm{jk}};\alpha_{\beta_{\mathrm{jk}}}\right)\times d_{\tau_{\mathrm{jk}}}\left(\tau_{\mathrm{jk}}|\alpha_{\beta_{\mathrm{jk}}}\right), \end{array}$$

where $d_{\beta_{\mathrm{jk}}}\left(\cdot \right)$ and $d_{\tau_{\mathrm{jk}}}\left(\cdot \right)$ refer to prior densities for $\beta_{\mathrm{jk}}$ and $\tau_{\mathrm{jk}}$, respectively. Further, each basis function l (l=1,…,L) used in the P‐splines has its own smoothing variance, denoted by $\tau_{\mathrm{ljk}}$. A commonly used prior density for $\tau_{\mathrm{ljk}}$ is the inverse gamma distribution (Umlauf, Klein, & Zeileis, 2018), given by

$$\begin{array}{c} d_{\tau_{\mathrm{ljk}}}\left(\tau_{\mathrm{ljk}}\right)\propto \tau_{\mathrm{jk}}^{‐(a+1)}\text{exp(}‐b/\tau_{\mathrm{jk}}), \end{array}$$

where a>0 and b>0 are the hyperparameters.

A commonly used prior density for $\beta_{\mathrm{jk}}$ is the density of a multivariate normal distribution (Umlauf *et al*., 2018), $N\left(m_{\mathrm{jk}},P_{\mathrm{jk}}{\left(\tau_{\mathrm{jk}}\right)}^{‐1}\right)$, where $m_{\mathrm{jk}}$ is the prior expectation and $P_{\mathrm{jk}}\left(\tau_{\mathrm{jk}}\right)$ is the prior precision matrix, which is equal to the inverse prior covariance matrix $\Sigma_{\mathrm{jk}}^{‐1}$.

In this paper, we will use the default inverse gamma density for $\tau_{\mathrm{jk}}$, and we will consider three different Gaussian priors for $\beta_{\mathrm{jk}}$: one weakly informative prior and two types of more strongly informative prior.

The weakly informative prior is based on a zero‐mean prior with precision matrix ${\tilde{P}}_{\mathrm{jk}}\left(\tau_{\mathrm{jk}}\right)=\tau_{\mathrm{jk}}^{‐2}K_{\mathrm{jk}}$, where $K_{\mathrm{jk}}$ is the P‐spline penalty matrix. This P‐spline penalty matrix defines the difference penalties on the coefficients of adjacent B‐splines (Eilers & Marx, 1996). A larger value of the smoothing parameter penalizes differences in coefficients more, yielding more smoothness in the estimated function. Imposing a smoothness penalty helps to prevent overfitting. The weakly informative prior expresses the smoothness assumption between the predictor(s) and the response variable, which makes the prior weakly informative. Thus, the weakly informative prior follows the $N(0,\tau_{\mathrm{jk}}^{2}K_{\mathrm{jk}}^{‐1})$ distribution. The models with weakly informative priors will be based on Markov chain Monte Carlo (MCMC) simulations.

The two more strongly informative priors (or informative priors for short) are based on a prior with mean $m_{\mathrm{jk}}$ and precision matrix ${\hat{P}}_{\mathrm{jk}}\left(\tau_{\mathrm{jk}}\right)$ based on the posterior mean (i.e., spline coefficients) and posterior precision matrix, respectively, of earlier data. Estimating these priors involves two stages: the analysis on the earlier data with the weakly informative prior as described before; and the analysis on new data with an informative prior based on the posterior of the first stage, using iteratively weighted least squares proposals (see Umlauf *et al*., 2018).

The first type of informative prior that we will use is a ‘posterior mode’ prior, defined as $N\left(m_{\mathrm{jk}},\tau_{\mathrm{jk}}^{2}{\hat{P}}_{\mathrm{jk}}{\left(\tau_{\mathrm{jk}}\right)}^{‐1}\right)$. We resort to maximizing the log‐posterior (an alternative way of estimating $\beta_{\mathrm{jk}}$ and $\tau_{\mathrm{jk}}$; Umlauf *et al*., 2018) because MCMC sampling is not possible when the posterior mean and posterior precision of the first stage as prior mean and prior precision are combined with additional constraints (i.e., the P‐spline penalty matrix of the second stage).

The second type of informative prior that we will use is a ‘fixed effects’ prior, defined as $N\left(m_{\mathrm{jk}},{\hat{P}}_{\mathrm{jk}}{\left(\tau_{\mathrm{jk}}\right)}^{‐1}\right)$, in which only the posterior mean and precision matrix from the first stage are used, without additional constraints. In this way, MCMC sampling is possible. We believe it makes sense theoretically to leave the additional constraints out because the first stage is already penalized and the smoothness of the function is already included in ${\hat{P}}_{\mathrm{jk}}\left(\tau_{\mathrm{jk}}\right)$. Also, by using the precision matrix from the first stage, it is prevented that the algorithm is only optimized in the direction of the second‐stage data.

## 3 Simulation study

The simulation study was performed in R (version 3.5.0; R Core Team, 2019). For the Bayesian distributional regression we used version 1.0‐2 of the *bamlss* package (Umlauf *et al*., 2018; Umlauf, Klein, Zeileis, & Simon, 2019). The R code and Data can be found on the Open Science Framework via https://osf.io/cjx3v/.

### 3.1 Research problem

In this simulation study we focus on efficiency and robustness. With regard to efficiency, we will investigate to what extent normed scores can be estimated more efficiently when including prior information. With regard to robustness, we will examine how robust the norm estimates are to prior misspecification; by ‘prior misspecification’ we mean a mismatch between the normative population distribution and the prior information. In addition, we will examine how the accuracy and precision of normed scores (i.e., percentiles) are influenced by four factors.

The first factor is the prior type and the second factor is the prior misspecification. For these factors, we expect the norm accuracy and precision to be better by using informative priors over weakly informative priors, with smaller and possibly opposite effects with larger prior misspecification. The third factor is the size of the normative sample on which the prior is based, denoted by $N_{\text{prior}}$. We only expect an effect for this factor when using informative priors, that is, that the norm estimations improve as $N_{\text{prior}}$ increases, with deteriorating effects for larger prior misspecifications. The fourth factor is the size of the normative sample for which the norms are estimated, denoted by $N_{\text{norm}}$. We expect the norm estimation to be better as $N_{\text{norm}}$ increases, and we expect the positive effect of including prior information to be relatively larger for small $N_{\text{norm}}$. The second factor relates to robustness, and the third and fourth factors relate to efficiency.

### 3.2 Design

**Figure 1:**
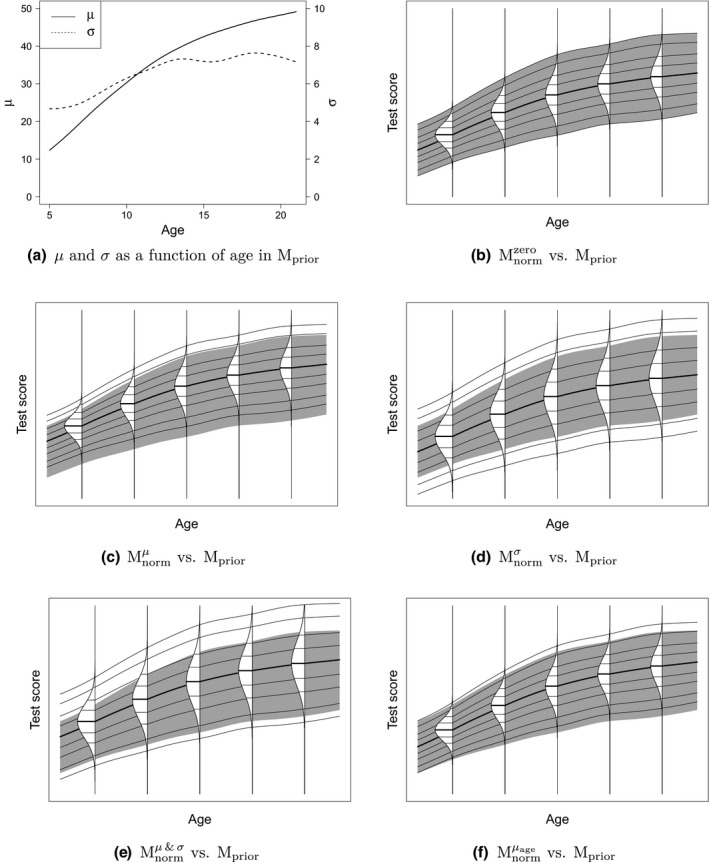
(a) Relationship between µ and σ, and age in $M_{\text{prior}}$. Shaded centile bands for $M_{\text{prior}}$ with centile curves and conditional PDFs of the five $M_{\text{norm}}$ models: (b) $M_{\text{norm}}^{\text{zero}}$, (c) $M_{\text{norm}}^{\mu }$, (d) $M_{\text{norm}}^{\sigma }$, (e) $M_{\text{norm}}^{\mu \&\sigma }$, (f) $M_{\text{norm}}^{\mu_{\text{age}}}$. The centile curves indicate percentiles 0.4, 2, 10, 25, 50, 75, 90, 98, and 99.6. The grey percentile bands in all panels indicate for $M_{\text{prior}}$ the range between the 0.4th and 99.6th percentiles of the test score distribution, conditional on age.

Two types of normative samples were generated in this simulation study: $Y_{\text{prior}}$ and $Y_{\text{norm}}$. The norming model estimated for $Y_{\text{prior}}$ was used as basis for the informative prior. The normed scores were estimated for $Y_{\text{norm}}$. To ensure that the simulation study is realistic, we based our population models on empirical normative data. The population model of $Y_{\text{prior}}$, denoted by $M_{\text{prior}}$, was a model estimated on German normative data from the composite ‘IQ Screening’ scale of the Intelligence and Developmental Scales 2 (IDS‐2; Grob & Hagmann‐von Arx, 2018). The IDS‐2 is a test for children and adolescents between 5 and 21 years of age, with norms dependent on age. Model $M_{\text{prior}}$ is the estimated Gaussian model on the empirical normative data, where the predictor age is related to distributional parameters µ (mean) and σ (standard deviation) using P‐splines. The ‘observed’ predictor values were taken as N equally spread values ranging from 5 to 21. The relationships of age with the mean (µ) and standard deviation (σ) of the Gaussian distribution in $M_{\text{prior}}$ are illustrated in Figure 1a.

The population model of $Y_{\text{norm}}$, denoted by $M_{\text{norm}}$, was similar to $M_{\text{prior}}$, with the degree of similarity between the two population models depending on the level of prior misspecification. The prior misspecification is defined as the difference between $M_{\text{norm}}$ and $M_{\text{prior}}$. The levels of prior misspecification were inspired by the difference in norming models as estimated on the German and Dutch (Grob, Hagmann‐von Arx, Ruiter, Timmerman, & Visser, 2018) normative data for the IDS‐2.

Four factors were systematically varied in a complete factorial design, with the number of levels between brackets:

1. prior type (3) – weakly informative, informative fixed effects, informative posterior mode;
2. prior misspecification (5) – zero, in µ, in σ, in µ and σ, age dependent in µ;
3. $N_{\text{prior}}$ (3) – 500, 1,000, 2,000;
4. $N_{\text{norm}}$ (3) – 250, 500, 1,000.

MCMC sampling was used for the models with the weakly informative prior and informative fixed effects prior. Samples were generated from two sequential Markov chains with 2,000 iterations each, of which 500 were for burn‐in.

The sample sizes $N_{\text{prior}}$ are in the typical range of what is being used in practice, and the range of values for $N_{\text{norm}}$ was chosen to be somewhat smaller than those for $N_{\text{prior}}$ to be able to check for efficiency. New samples $Y_{\text{prior}}$ were generated for each level of $N_{\text{prior}}$, with R=1,000 replications each, which resulted in 3 ($N_{\text{prior}}$) × 1,000 (*R*) = 3,000 generated data sets. New samples $Y_{\text{norm}}$ were generated for each level of $N_{\text{prior}}$, $N_{\text{norm}}$ and prior misspecification, also with 1,000 replications each, which resulted in 3 ($N_{\text{prior}}$) × 3 ($N_{\text{norm}}$) × 5 (prior misspecification) × 1,000 (*R*) = 45,000 generated data sets.

To be able to use the spline coefficients as prior information, the number of knots of the P‐splines was held constant at 24 across all models. Eilers and Marx (2010) recommended using equally spaced knots. The number of knots must be high enough to fit features in the data, but after this minimum number has been reached, additional knots have little effect on the fit (Ruppert, 2002). The optimal number of knots and their location were determined for $M_{\text{prior}}$. Using the corrected Akaike information criterion (AICc), it was determined that using 24 knots was optimal. The number of knots and the knot locations were taken equal for both distributional parameters (µ and σ), which means that both distributional parameters have *J* functions relating the regression parameters $\beta_{\mathrm{jk}}$ and the predictor.

The different levels of prior misspecification are illustrated in Figure 1. The grey shading in Figure 1b–f indicates for $M_{\text{prior}}$ the range between the extreme percentiles (i.e., 0.4th and 99.6th), conditional on age. Also shown are centile curves and conditional PDFs in the same percentile range, with centile curves, conditional on age.

The centile curves and conditional PDFs in Figure 1b–f correspond to $M_{\text{norm}}$. If the prior is misspecified, it can be misspecified in many ways. We look at Gaussian priors with a shift in µ and/or σ. The difference between the centile curves and grey shading illustrates the five different levels of prior misspecification in the simulation study: zero misspecification (b), a misspecification in µ (c), in σ (d), in µ and σ (e), and an age‐dependent misspecification in µ (f). The corresponding population models are denoted by $M_{\text{norm}}^{\text{zero}}$, $M_{\text{norm}}^{\mu }$, $M_{\text{norm}}^{\sigma }$, $M_{\text{norm}}^{\mu \&\sigma }$, and $M_{\text{norm}}^{\mu_{\text{age}}}$, respectively. The differences in distributional parameters (i.e., µ and σ) between the population models can be found in Table S1.

#### 3.2.1 Outcome measures

The convergence of the Markov chains was investigated with the potential scale reduction factor ($\hat{R}$; Gelman & Rubin, 1992) for each parameter. $\hat{R}$ is the factor by which the scale of the distribution for the estimated parameter might be reduced by running the chains longer. The closer $\hat{R}$ is to 1, the smaller the potential scale reduction. Using the rule of thumb proposed by Gelman *et al*. (2014), we assumed sufficient convergence whenever $\hat{R}$ < 1.1.

To express the estimated accuracy and precision, we consider the population and model‐implied conditional distributions. We express this difference as the root mean square error (RMSE) – which captures both accuracy and precision – by marginalizing out both age and the test score. The smaller the RMSE value, the smaller the discrepancy between the estimated and true percentiles over all ages and test scores. To marginalize out age and test score, we numerically approximated the integral by evaluating the estimated percentiles ($\hat{\theta }$) and the true percentiles (θ) at *X* = 1,000 equally spaced age values *x* across the full age range [5, 21] and *Y* = 1,000 test scores *y* corresponding to true *z* scores in the range [−3, +3], conditional on *X*. Conditional test scores outside this range (i.e., deviating more than 3 standard deviations from the mean score) are not reported in practice (e.g., in the IDS‐2) because the uncertainty in those scores is considered to be too large and therefore not relevant in our outcome measure. Thus, the RMSE is calculated as

$$\text{RMSE}\;=\;\sqrt{\frac{1}{\mathrm{XY}}\sum_{i=1}^{X}\sum_{j=1}^{Y}{\left({\hat{\theta }}_{x_{i}y_{j}}‐\theta_{x_{i}y_{j}}\right)}^{2}.}$$

## 4 Results

For the convergence of the Markov chains, we considered convergence to be sufficient for those chains with $\hat{R}<1.1$. Inspection of the 97.5th quantile of the $\hat{R}$ for all spline coefficients of all 1,000 replications across all conditions showed good convergence, with almost all $\hat{R}$ values below 1.1: for only 0.07% of all estimated spline coefficients they were 1.1 or greater. Across all combinations of $N_{\text{prior}}$, $N_{\text{norm}}$, prior misspecification, and prior type separately, the proportion of $\hat{R}$ greater than or equal to 1.1 ranged from 0% to 0.197%. Keeping the other factors constant, $\hat{R}$ increased as $N_{\text{norm}}$ decreased. Furthermore, $\hat{R}$ was larger for the weakly informative prior than for the fixed effects informative prior, given the other factors, which indicates that model estimation with the latter was more efficient.

To obtain insight into the relative effects of the factors on the RMSE, a full‐factorial mixed effects analysis of variance (ANOVA) was performed. $N_{\text{prior}}$ was a between factor, $N_{\text{norm}}$ and the prior misspecification were within factors, and the prior type was nested within the within factors. We included the main effects and all higher‐order interactions in the model, but we were specifically interested in the main effects. Results are provided in Table S2. The ANOVA results indicate that the relative effects of the prior misspecification and of the norm sample size ($N_{\text{norm}}$) on the RMSE is largest ($\omega^{2}$ = .206 and .149, respectively), and the relative effect of $N_{\text{prior}}$ on the RMSE is smallest ($\omega^{2}$ = .005).

### 4.1 Root mean square error

**Figure 2:**
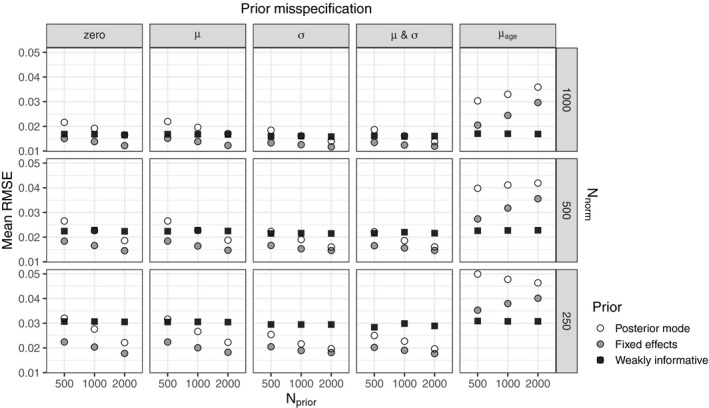
Plots with the mean RMSE across all combinations of prior type, prior misspecification, $N_{\text{prior}}$, and $N_{\text{norm}}$.

The mean RMSEs across 1,000 replications of all conditions are shown in Figure 2 and, with the standard deviations, in Table S3. The standard error of the mean RMSE varies from 9.0 × 10^−5^ to 5.8 × 10^−4^ across all conditions. The results show that the informative posterior mode prior is outperformed by the informative fixed effects prior and/or the weakly informative prior within all conditions. That is why we focus on the results of the informative fixed effects prior and weakly informative prior only.

When there was no prior misspecification, the mean RMSE of the informative fixed effects prior was consistently lower than the mean RMSE of the weakly informative prior. Regardless of prior type, the mean RMSE decreased as $N_{\text{norm}}$ increased. For the informative prior, the mean RMSE decreased as $N_{\text{prior}}$ increased, while it did not depend on $N_{\text{prior}}$ for the weakly informative prior, as could be expected. Similar patterns were found when there was an age‐independent prior misspecification, in µ, in σ, and in both µ and σ.

When there was an age‐dependent prior misspecification in µ, denoted by $\mu_{\text{age}}$, the weakly informative prior outperformed the informative fixed effects prior, regardless of $N_{\text{prior}}$ and $N_{\text{new}}$. In contrast to the other levels of prior misspecification, the mean RMSE of the informative fixed effects prior *increased* as $N_{\text{prior}}$ increased. There was again no effect of $N_{\text{prior}}$ on the mean RMSE for the weakly informative prior. Similarly to the other levels of prior misspecification, the mean RMSE decreased as $N_{\text{norm}}$ increased, regardless of prior type.

### 4.2 Interpretation of root mean square error

To give an idea of the interpretation of the size of the RMSE values, we show the difference between true and estimated centile curves.

**Figure 3:**
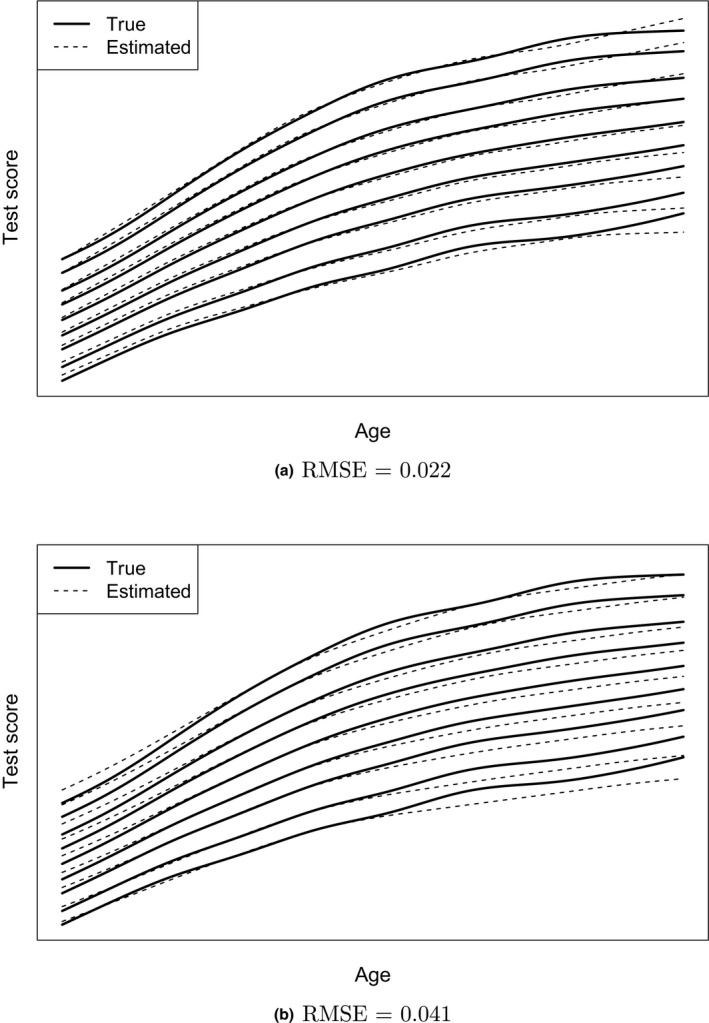
Centile curves for the population model (solid lines) and the estimated model (dashed lines) for one replication of two conditions differing in $N_{\text{prior}}$ and $N_{\text{norm}}$: (a) condition 1, with RMSE value of 0.022, has $N_{\text{prior}}=500$ and $N_{\text{norm}}=1,000$; (b) condition 2, with RMSE value of 0.041, has $N_{\text{prior}}=2,000$ and $N_{\text{norm}}=250$. Both conditions have an age‐dependent prior misspecification in µ, and the fixed effects prior. The centile curves represent percentiles 0.4, 2, 10, 25, 50, 75, 90, 98, and 99.6.

Figure 3 shows for one replicate how the estimated centiles curves (dashed lines) deviate from the population centile curves (solid lines). Both conditions have $M_{\text{norm}}^{\mu_{\text{age}}}$ and the fixed effects prior, but they differ in $N_{\text{prior}}$ and $N_{\text{norm}}$. Figure 3a denotes a replication with a relatively low RMSE value of 0.022, with $N_{\text{prior}}$ equal to 500 and $N_{\text{norm}}$ equal to 1,000. Figure 3b depicts a relatively high RMSE value of 0.041, with $N_{\text{prior}}$ equal to 2,000 and $N_{\text{norm}}$ equal to 250. The difference in RMSE values can be clearly seen.

The overall deviation is quite small for the middle of the age range and largest for the highest age values. The influence of the age‐dependent misspecified prior is larger in Figure 3b than in Figure 3a because $N_{\text{prior}}$ is larger and $N_{\text{norm}}$ is smaller. The deviation in Figure 3b resembles the difference in centile curves under $M_{\text{prior}}$ and $M_{\text{norm}}^{\mu_{\text{age}}}$ as shown in Figure 1f.

## 5 Application of Bayesian Gaussian norm estimation to the IDS‐2 normative data

We illustrate the use of prior information in norm estimation with Gaussian models using empirical normative data of the German and Dutch IDS‐2 (Grob *et al*., 2018). The R code for this procedure is available as Code S1. In this illustration we estimate the percentiles of the composite ‘IQ Screening’ scale for the normative data from the Dutch IDS‐2 ($N_{\text{norm}}=1,566$), with prior information based on the normative data from the German IDS‐2 ($N_{\text{prior}}=1,652$). We have no theoretical reasons (e.g., related to the education system) to assume that the population models underlying the normed scores of this scale substantially differ across the two countries.

**Figure 4:**
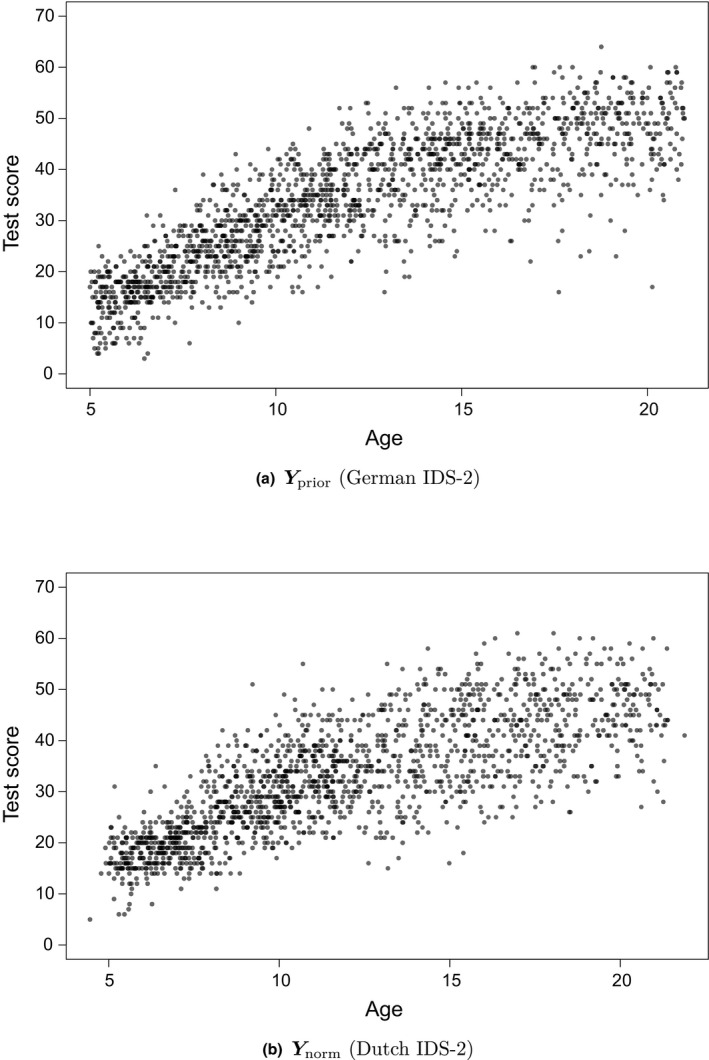
Scatter plots showing the relationship between the test scores and age for (a) $Y_{\text{prior}}$ and (b)$Y_{\text{norm}}$, which are the empirical normative data of the German IDS‐2 and Dutch IDS‐2, respectively.

Inspection of the relationship between the raw test scores and age for both samples ($Y_{\text{prior}}$ and $Y_{\text{norm}}$), in Figure 4a,b respectively, reveals that this relationship looks similar for both samples. The spread of the scores seems somewhat larger for $Y_{\text{norm}}$ than for $Y_{\text{prior}}$, but this could be due to sampling fluctuations. Based on theoretically based expectations and visual comparison, we presume that possible prior misspecification is of a minor nature.

We compare the estimated models based on the weakly informative prior and the fixed effects informative prior. We refrain from considering the posterior mode prior, because it consistently performed worse than the other two priors in our simulation study. We use a Gaussian model with P‐splines to model the relationship between the test score distribution and age. Using the AICc as a criterion indicates the use of 24 equally spaced knots. We first estimate the Gaussian model on $Y_{\text{prior}}$ and extract the posterior mean (spline coefficients), posterior precision matrix and knot locations. The posterior mean and posterior precision matrix are then used as prior mean and prior precision matrix in estimating the model with the fixed effects prior on $Y_{\text{norm}}$, using the same knot locations. Note that the age range in $Y_{\text{norm}}$ should not be outside the inner knot range based on $Y_{\text{prior}}$. Because 23 observations of $Y_{\text{norm}}$ had age values slightly outside this range of $\left[4.984,21.016\right]$, we forced them to be equal to the bounds of this range.

**Figure 5:**
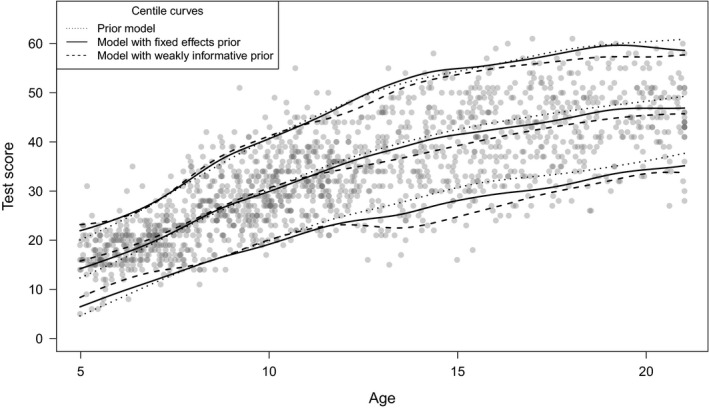
Centile curves (5th, 50th and 95th percentiles) corresponding to the estimated prior model (dotted line), model with fixed effects informative prior (solid line), and model with weakly informative prior (dashed line). The dots indicate the observations of $Y_{\text{norm}}$.

Figure 5 shows the centile curves (5th, 50th and 95th percentiles) corresponding to the estimated prior model (dotted line), model with fixed effects prior (solid line), and model with weakly informative prior (dashed line). The dots indicate the observations of $Y_{\text{norm}}$. The results show that the centile curves of the three models overlap in the range 8–12 years and are further apart outside this range. In general, conditional on a percentile, the centile curves of the model with the fixed effects informative prior lie between the centile curves of the other two models. This makes sense, because this model is a combination of the prior model and $Y_{\text{norm}}$, on which the model with the weakly informative prior is heavily based. The centile curve of the 5th percentiles for the model with weakly informative prior seems to be heavily pulled towards the outliers around age 14.

**Figure 6:**
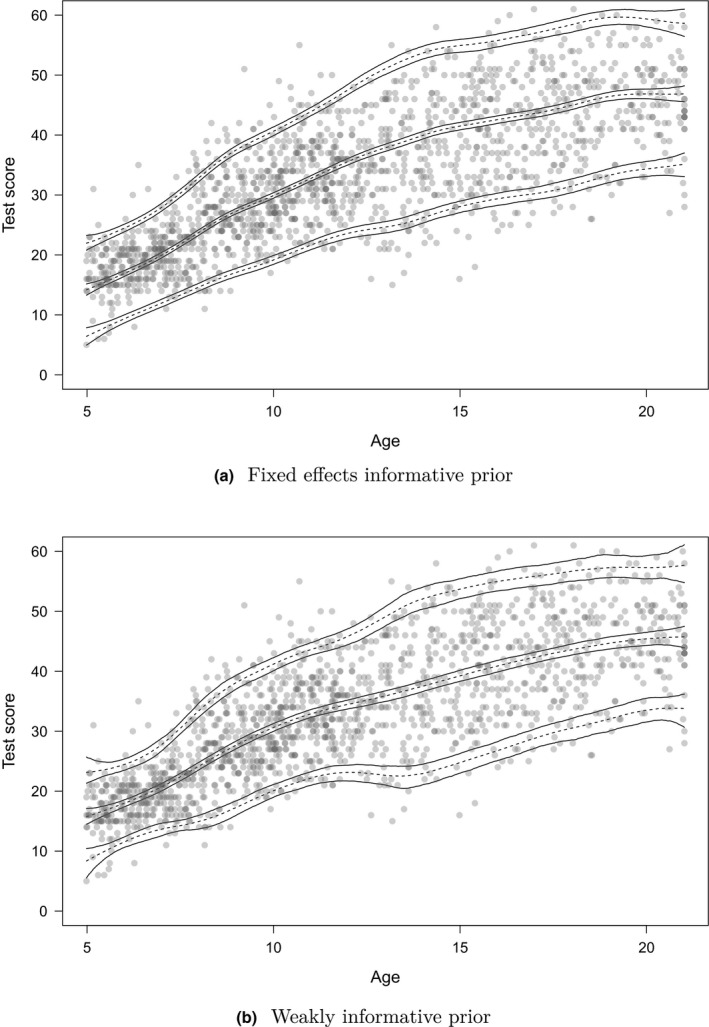
The posterior mean (dashed line) and 95% credible intervals (solid lines) of the posterior distribution of the 5th, 50th and 95th percentiles as a function of age, based on 1,001 samples of the posterior distribution of µ and σ as a function of age, for (a) the model with the fixed effects informative prior, and (b) the model with the weakly informative prior.

Figure 6 shows the posterior mean and the 95% credible intervals of the posterior distribution of the 5th, 50th and 95th percentiles as a function of age, based on 1,001 samples of the posterior distribution of µ and σ as a function of age, for the model with the fixed effects informative prior and the model with the weakly informative prior. This shows that the percentile estimates have more precision when estimated with the fixed effects informative prior than with the weakly informative prior. In addition, this figure shows that the estimates of the extreme percentiles (i.e., 5th and 95th percentiles) are less precise than the estimates of the median, and the percentile estimates near the boundaries of the predictor space are less precise than those in the middle of the predictor space.

## 6 Discussion

The results of the simulation study showed that for the simulated prior misspecification the normed scores (i.e., percentiles) could be estimated more efficiently by using prior information, as long as the prior misspecification was not age‐dependent. The performance under fixed effects informative priors was better than under the posterior mode informative priors, even when there was no prior misspecification. The use of proper prior information yielded a substantial gain in efficiency. For example, in the condition with no prior misspecification and a prior sample size of 2,000, an $N_{\text{norm}}$ of 250 resulted in about the same mean RMSE as not using prior information with an $N_{\text{norm}}$ of 1,000.

As expected, $N_{\text{prior}}$ only had an effect on the mean RMSE when using informative priors, with better norm estimations for increasing $N_{\text{prior}}$. This effect did not seem to be affected by the level of prior misspecification. Also, the norm estimation was better for increasing $N_{\text{norm}}$, and the added value of including prior information was larger for small $N_{\text{norm}}$.

The results were robust against relatively large prior misspecifications in the mean and standard deviation of the conditional score distribution, when these misspecifications were age independent. Even with an age‐dependent prior misspecification and small $N_{\text{norm}}$, the overall centile curves were retrieved quite well. We evaluated the discrepancy between the true and estimated percentiles over a range of scores and age values, but the prior misspecification is likely to vary locally. So, also for age‐dependent prior misspecification, the percentiles might be estimated well for some age and score ranges, but worse for other ranges.

In practice, the level of prior misspecification is unknown. If there are theoretical reasons to believe that in the population the relationship between the (sub)test scores and the predictor is different in another country, and/or if inspection of the normative sample indicates a completely different relationship, we advise against using prior information. We did not test for age‐dependent prior misspecifications in σ, but we suspect that using prior information deteriorates the norm estimation in that situation as well.

A practically useful approach seems to be to collect a relatively small normative sample (e.g., N=250), and then check whether it might be reasonable to assume that the normative sample and the prior sample have the same relationship between the distributional parameters and predictor. Then, it is decided based on this whether prior information can be used or whether it is necessary to continue sampling. Our recommendation for future research is to develop diagnostics that help to determine to what extent prior information can be used in the creation of new norms.

A limitation of this study is that we only used Gaussian models. In norming practice (e.g., Grob *et al*., 2018; Voncken *et al*., 2018), we often deal with non‐normality, which requires more flexible models. The scatter plot of $Y_{\text{prior}}$ in Figure 4a suggests that the conditional score distribution is negatively skewed, which might be modelled better with a skew normal distribution. The proposed method is applicable to different and additional distributional parameters (e.g., a skewness parameter) as well. This proof of concept based on the Gaussian model shows that including prior information can make norm estimation more efficient, so it is important for future research to investigate the performance of this method for other distributions as well.

An additional suggestion for future research is to explore the use of monotonic P‐splines in combination with prior norm information. When the mean (or median) test score is theoretically expected to increase with age, monotonic P‐splines can be used to force a monotonically increasing relationship between the location parameter (i.e., µ) and age. In this way, theoretical expectations can be incorporated and the sampling variability can be reduced further. While Bayesian monotonic P‐splines are currently not yet implemented in the *bamlss* R package, previous research has shown that they can be successfully applied (Brezger & Steiner, 2008).

A general limitation of standard regression models is that they do not accommodate measurement errors in the predictors (Carroll, Ruppert, Stefanski, & Crainiceanu, 2006). Variables that are typically used as predictors in psychological test norming, such as age, sex and education level, are relatively easy to measure, and are unlikely to be prone to measurement errors due to a measurement device. While it is theoretically possible to use the exact values of continuous predictors, they have to be discretized (and rounded) in practice, which introduces some discretization error. In our study, age was rounded to six decimal places. We expect the possible bias because of this internal rounding to be very small (see, for example, Lang, Umlauf, Wechselberg, Harttgen, & Kneib, 2012). When the measurement error is expected to be more severe (i.e., due to the measurement itself), one could correct for this error by following the ideas developed in Pollice *et al*. (2019).

In conclusion, using prior information in norm estimation can be useful. In the norming context we often have prior information available in the form of the previous normative sample scores of the test or normative sample scores in a different country. When we have theoretical and empirical reasons to assume that the relationship between the test score distribution and the predictor is similar in the population, the same norm precision can be achieved with a much smaller normative sample. This helps test developers to achieve cost‐efficient high‐quality norms.

## Conflicts of interest

All authors declare no conflict of interest.

## Author contributions

Lieke Voncken (Conceptualization; Formal analysis; Methodology; Writing – original draft; Writing – review & editing) Thomas Kneib (Conceptualization; Methodology; Software; Writing – review & editing) Casper J. Albers (Conceptualization; Methodology; Supervision; Writing – review & editing) Nikolaus Umlauf (Methodology; Software; Writing – review & editing) Marieke E. Timmerman (Conceptualization; Methodology; Supervision; Writing – review & editing.

## Supporting information


**Table S1.** Distributional parameters of the population models in the simulation study.
**Table S2.** Values of ω^2^ from full‐factorial mixed effects ANOVA on the RMSE.
**Table S3.** Mean RMSEs (and SDs) of the models across prior type, prior misspecification, *N*_prior_, and *N*_norm_, across 1,000 replications.

**Data S1.** R code empirical illustration.

## Data Availability

The simulated data that support the findings of this study can be reproduced with the simulation R code, which is openly available via the Open Science Framework (OSF) at https://osf.io/cjx3v/. The empirical normative data in the illustration were used under license for this study. Example simulated data based on this empirical data are openly available via the same OSF link.
