# Supplementary material for: Bayesian Gaussian distributional regression models for more efficient norm estimation
Source: Br J Math Stat Psychol. 2020 Jul 20;74(1):99–117. doi: 10.1111/bmsp.12206 (PMC7891623; doi:10.1111/bmsp.12206)
Supplement: Supplementary file 1 — Table S1. Distributional parameters of the population models in the simulation study. Table S2. Values of ω2 from full‐factorial mixed effects ANOVA on the RMSE. Table S3. Mean RMSEs (and SDs) of the models across prior type, prior misspecification, N prior, and N norm, across 1,000 replications. [file BMSP-74-99-s001.pdf]

Table S1: Distributional parameters of the population models in the simulation study

| | | Distributional parameter | |
|---|---|---|---|
| Population model | | $\mu$ | $\sigma$ |
| $\mathrm{M}_{\mathrm{prior}}$ | | $g(\mathrm{age})$ | $h(\mathrm{age})$ |
| $\mathrm{M}_{\mathrm{norm}}$ | zero | $g(\mathrm{age})$ | $h(\mathrm{age})$ |
| | $\mu$ | $g(\mathrm{age}) + 5$ | $h(\mathrm{age})$ |
| | $\sigma$ | $g(\mathrm{age})$ | $h(\mathrm{age}) + 3$ |
| | $\mu$ & $\sigma$ | $g(\mathrm{age}) + 5$ | $h(\mathrm{age}) + 3$ |
| | $\mu_{\mathrm{age}}$ | $1.1\, g(\mathrm{age}) - 10$ | $h(\mathrm{age})$ |

*Note.* $\mathrm{M}_{\mathrm{prior}}$ and $\mathrm{M}_{\mathrm{norm}}$ indicate the population models, with distributional parameters $\mu$ (mean) and $\sigma$ (standard deviation). $g(\mathrm{age}) = \boldsymbol{\beta_{\mu 0}} + \boldsymbol{\beta_{\mu 1}} \cdot f(\mathrm{age})$, and $h(\mathrm{age}) = \boldsymbol{\beta_{\sigma 0}} + \boldsymbol{\beta_{\sigma 1}} \cdot f(\mathrm{age})$.

Table S2: Values of $\omega^2$ from full-factorial mixed effects ANOVA on the RMSE

| | $\omega^2$ | *SS* | *df* |
|---|---|---|---|
| **$N_{prior}$** | **.005** | **0.083** | **2** |
| **$N_{norm}$** | **.149** | **2.253** | **2** |
| $N_{prior}$ : $N_{norm}$ | .001 | 0.014 | 4 |
| **prior misspecification** | **.206** | **3.118** | **4** |
| $N_{prior}$ : prior misspecification | .009 | 0.139 | 8 |
| $N_{norm}$ × prior misspecification | .006 | 0.091 | 8 |
| $N_{prior}$ : $N_{norm}$ : prior misspecification | .001 | 0.010 | 16 |
| **prior type** | **.050** | **0.754** | **2** |
| $N_{prior}$ : prior type | .007 | 0.105 | 4 |
| $N_{new}$ : prior type | .010 | 0.155 | 4 |
| prior misspecification : prior type | .096 | 1.450 | 8 |
| $N_{prior}$ : $N_{norm}$ : prior type | .001 | 0.012 | 8 |
| $N_{prior}$ : prior misspecification : prior type | .005 | 0.072 | 16 |
| $N_{norm}$ × prior misspecification : prior type | .003 | 0.044 | 16 |
| $N_{prior}$ : $N_{norm}$ × prior misspecification : prior type | <.001 | 0.007 | 32 |
| Residual | - | 6.817 | 134,865 |

*Note.* The main effects are displayed in bold font. The colon indicates an interaction effect with nesting of the factor on the right within the factor on the left. The cross indicates an interaction effect of factors with the same nesting level.

Table S3: Mean RMSEs (and SDs) of the models across prior type, prior misspecification, $N_{\text{prior}}$, and $N_{\text{norm}}$, across 1,000 replications.

| Prior misspecification | | | zero | | | $\mu$ | | | $\sigma$ | | | $\mu$ & $\sigma$ | | | $\mu_{\text{age}}$ | | |
|---|---|---|---|---|---|---|---|---|---|---|---|---|---|---|---|---|---|
| $N_{\text{orig}}$ | $N_{\text{norm}}$ | Prior | PM | FE | WI | PM | FE | WI | PM | FE | WI | PM | FE | NI | PM | FE | WI |
| 500 | 250 | | 3.206 | 2.242 | 3.070 | 3.166 | 2.240 | 3.05 | 2.543 | 2.047 | 2.952 | 2.504 | 2.022 | 2.840 | 4.996 | 3.532 | 3.091 |
| | | | (1.094) | (0.624) | (0.809) | (0.705) | (0.665) | (0.864) | (0.635) | (0.635) | (0.840) | (0.631) | (0.609) | (0.826) | (1.161) | (0.727) | (0.814) |
| | 500 | | 2.654 | 1.837 | 2.242 | 2.655 | 1.842 | 2.239 | 2.231 | 1.664 | 2.149 | 2.217 | 1.653 | 2.158 | 3.979 | 2.738 | 2.260 |
| | | | (0.582) | (0.465) | (0.595) | (0.595) | (0.472) | (0.588) | (1.224) | (0.476) | (0.584) | (1.206) | (0.441) | (0.585) | (0.720) | (0.604) | (0.573) |
| | 1,000 | | 2.162 | 1.507 | 1.684 | 2.197 | 1.514 | 1.682 | 1.845 | 1.334 | 1.594 | 1.866 | 1.348 | 1.617 | 3.036 | 2.050 | 1.704 |
| | | | (0.444) | (0.364) | (0.413) | (0.466) | (0.342) | (0.391) | (0.759) | (0.329) | (0.398) | (0.998) | (0.328) | (0.405) | (0.997) | (0.450) | (0.412) |
| 1,000 | 250 | | 2.764 | 2.036 | 3.070 | 2.669 | 2.007 | 3.056 | 2.165 | 1.890 | 2.950 | 2.272 | 1.905 | 2.988 | 4.779 | 3.798 | 3.077 |
| | | | (1.821) | (0.619) | (0.811) | (1.049) | (0.600) | (0.785) | (0.621) | (0.605) | (0.828) | (1.468) | (0.650) | (0.843) | (1.088) | (0.637) | (0.817) |
| | 500 | | 2.264 | 1.659 | 2.282 | 2.272 | 1.641 | 2.277 | 1.900 | 1.529 | 2.160 | 1.860 | 1.560 | 2.201 | 4.112 | 3.178 | 2.271 |
| | | | (0.497) | (0.436) | (0.587) | (0.985) | (0.439) | (0.605) | (1.388) | (0.418) | (0.572) | (0.462) | (0.451) | (0.578) | (0.623) | (0.537) | (0.589) |
| | 1,000 | | 1.919 | 1.382 | 1.678 | 1.958 | 1.383 | 1.678 | 1.613 | 1.257 | 1.603 | 1.618 | 1.242 | 1.589 | 3.298 | 2.445 | 1.700 |
| | | | (0.409) | (0.329) | (0.415) | (0.957) | (0.336) | (0.405) | (1.001) | (0.316) | (0.420) | (1.000) | (0.286) | (0.384) | (0.996) | (0.447) | (0.420) |
| 2,000 | 250 | | 2.215 | 1.778 | 3.056 | 2.227 | 1.823 | 3.045 | 1.964 | 1.813 | 2.948 | 1.961 | 1.770 | 2.892 | 4.638 | 4.014 | 3.077 |
| | | | (1.065) | (0.633) | (0.828) | (0.621) | (0.656) | (0.798) | (0.950) | (0.649) | (0.851) | (1.120) | (0.603) | (0.785) | (0.556) | (0.501) | (0.814) |
| | 500 | | 1.862 | 1.449 | 2.237 | 1.879 | 1.470 | 2.250 | 1.599 | 1.457 | 2.149 | 1.605 | 1.462 | 2.160 | 4.190 | 3.557 | 2.278 |
| | | | (0.424) | (0.409) | (0.558) | (0.443) | (0.433) | (0.580) | (0.420) | (0.423) | (0.556) | (0.430) | (0.424) | (0.593) | (1.001) | (0.444) | (0.585) |
| | 1,000 | | 1.650 | 1.218 | 1.651 | 1.706 | 1.225 | 1.674 | 1.403 | 1.169 | 1.582 | 1.379 | 1.194 | 1.607 | 3.589 | 2.965 | 1.689 |
| | | | (0.943) | (0.294) | (0.398) | (1.553) | (0.310) | (0.397) | (1.206) | (0.284) | (0.389) | (0.741) | (0.312) | (0.414) | (1.304) | (0.391) | (0.401) |

*Note.* SDs between parentheses. All RMSEs are multiplied by 100. PM, FE, and WI represent the posterior mode, fixed effects, and weakly informative prior, respectively. $\mu$, $\sigma$, $\mu$ & $\sigma$, and $\mu_{\text{age}}$ represent an age (in)dependent prior misspecification in $\mu$, $\sigma$, or both.
