# Supplementary material for: Bayesian Gaussian distributional regression models for more efficient norm estimation
Source: Br J Math Stat Psychol. 2020 Jul 20;74(1):99–117. doi: 10.1111/bmsp.12206 (PMC7891623; doi:10.1111/bmsp.12206)
Supplement: Supplementary file 2 — Data S1. R code empirical illustration. [file BMSP-74-99-s002.docx]

### Supplemental Material

# "Bayesian Gaussian distributional regression models for more efficient norm estimation"

# R code to use prior information in norm estimation

# Use with files "examplepriordata.txt" and "examplenormingdata.txt".

###############################################################

##################### (1) Initialisation ######################

###############################################################

# =========================================================== #

# (a) Packages #

# =========================================================== #

install.packages("bamlss", repos="http://R-Forge.R-project.org")

library(bamlss)

# For "alpha" function

install.packages("scales")

library(scales)

install.packages("MASS")

library(MASS) # for "ginv" function

set.seed(1234)

# =========================================================== #

# (b) Read data #

# =========================================================== #

mypriordata <- read.table("examplepriordata.txt", header = T)

mynormdata <- read.table("examplenormingdata.txt", header = T)

summary(mypriordata)

summary(mynormdata)

# Select your own variables. Make dataframe with names "y" and "age"

priordata <- data.frame(y = mypriordata$y, age = mypriordata$age)

normdata <- data.frame(y = mynormdata$y, age = mynormdata$age)

# Make sure that both dataframes have the same predictor range (or at least that the predictor range in the normdata is not outside the predictor range in the priordata).

normdata$age <- ifelse(normdata$age < min(priordata$age), min(priordata$age), normdata$age)

normdata$age <- ifelse(normdata$age > max(priordata$age), max(priordata$age), normdata$age)

par(mfrow = c(1,2))

plot(priordata$age, y = priordata$y, xlab = "Age", ylab = "Test score", cex.lab = 1.5, col = scales::alpha("black", 0.6), pch = 20, cex.axis = 1.2, main = "priordata")

plot(normdata$age, y = normdata$y, xlab = "Age", ylab = "Test score", cex.lab = 1.5, col = scales::alpha("black", 0.6), pch = 20, cex.axis = 1.2, main = "normdata")

###############################################################

################## (2) Estimate prior model ###################

###############################################################

f_prior <- list(

y ~ s(age, bs="ps", k=20), # using your own optimal number of knots

sigma ~ s(age, bs="ps", k=20)

)

mod_prior <- bamlss(f_prior, data = priordata, family = "gaussian")

## Posterior mean of mu and sigma

pm <- coef(mod_prior, FUN = mean, hyper.parameters = FALSE, pterms = FALSE, list = TRUE)

# Posterior precision of mu and sigma

pms <- coef(mod_prior, FUN = function(x) { x }, hyper.parameters = FALSE, pterms = FALSE, list = TRUE)

pS.mu <- ginv(cov(t(pms$mu)))

pS.sigma <- ginv(cov(t(pms$sigma)))

## Extract knots.

kn <- mod_prior$x$mu$smooth.construct[["s(age)"]]$knots

###############################################################

############## (3) Estimate model for new sample ###############

###############################################################

# Use the informative fixed effects prior, and the weakly informative prior

# Fixed effects # specify the prior mean and prior precision based on mod_prior

f_norm_FE <- list(

y ~ s(age, bs="ps", k=20, xt=list("pm"=pm$mu,"pS"=pS.mu), fx=TRUE),

sigma ~ s(age, bs="ps", k=20, xt=list("pm"=pm$sigma,"pS"=pS.sigma), fx=TRUE)

)

mod_norm_FE <- bamlss(f_norm_FE, data = normdata, family = "gaussian", propose = "iwls", knots = list("age" = kn))

# Weakly informative prior

f_norm_WI <- list(

y ~ s(age, bs = "ps", k=20),

sigma ~ s(age, bs = "ps", k=20)

)

mod_norm_WI <- bamlss(f_norm_WI, data = normdata, family = "gaussian", k = list("age" = kn))

###############################################################

############# (4) Derive estimated centile curves #############

###############################################################

## Function for drawing centile curves

curves.func <- function(mod = mod_prior, col = "black", lty = 1){

# Age values in plot

pop.age <- seq(min(normdata$age), max(normdata$age), length.out = 1000)

# Percentiles

p <- c(0.05, 0.50, 0.95)

# Predict distributional parameters for those age values

mu.new <- predict(mod, model = "mu", newdata = data.frame(age = pop.age))

sigma.new <- exp(predict(mod, model = "sigma", newdata = data.frame(age = pop.age)))

est.perc <- matrix(NA, ncol = length(p), nrow = length(pop.age))

for (i in 1:length(pop.age)){

est.perc[i,] <- qnorm(p, mean = mu.new[i], sd = sigma.new[i])

}

for (j in 1:length(p)){

lines(est.perc[,j], x = pop.age, lwd = 2, col = col, lty = lty)

}

}

par(mar=c(5.1,5,4.1,2.1))

# First draw empty plot

par(mfrow = c(1,1))

plot(0, xlim = c(5, 21), ylim = c(0, max(normdata$y)+5), ylab = "Test score", xlab = "Age", cex.lab = 1.5, cex.axis = 1.2)

# Add observations

points(normdata$y, x = normdata$age, pch = 20, col = scales::alpha("grey34", 0.3), cex = 1.5)

curves.func(mod = mod_prior, col = "black", lty = 3)

curves.func(mod = mod_norm_FE, col = "black", lty = 1)

curves.func(mod = mod_norm_WI, col = "black", lty = 2)

###############################################################

###### (5) Find percentiles for score conditional on age ######

###############################################################

### Derive percentile for a score conditional on a specific age value.

age.percentile <- 15

score.percentile <- 20

est.mu <- predict(mod_norm_FE, model = "mu", newdata = data.frame(age = age.percentile))

est.sigma <- exp(predict(mod_norm_FE, model = "sigma", newdata = data.frame(age = age.percentile)))

# Vector of test scores with the same range as in the sample

y.uniform <- seq(from = min(normdata$y), to = max(normdata$y), length.out = 3000)

# Compute percentiles for the range of test scores, conditional on the specified age value

cdf.vector <- pnorm(y.uniform, mean = est.mu, sd = est.sigma)

# Compute percentiles for the specified test score, conditional on the specified age value

found.percentile <- pnorm(score.percentile, mean = est.mu, sd = est.sigma)

plot(cdf.vector, x = y.uniform, ylab = "CDF", xlab = paste("Test score | age = ",age.percentile, sep = ""), type = "l", lwd = 1)

segments(x0 = score.percentile, y0 = 0, x1 = score.percentile, y1 =

found.percentile, lty = 2)

segments(x0 = 0, x1 = score.percentile, y0 = found.percentile, y1 =

found.percentile, lty = 2)

found.percentile
